# Experiential Avoidance and Youth Mental Health: The Role of Spontaneity and Creative Adaptability

**DOI:** 10.3390/healthcare14142084

**Published:** 2026-07-12

**Authors:** Alexandra Banciu, Camelia Soponaru, Mihaela Dana Bucuță

**Affiliations:** 1 Faculty of Psychology and Education Sciences, “Alexandru Ioan Cuza” University, 700554 Iasi, Romania; camelia.soponaru@uaic.ro; 2 Department of Psychology, Faculty of Social Sciences and Humanities, “Lucian Blaga” University, 550024 Sibiu, Romania

**Keywords:** experiential avoidance, spontaneity, creative adaptability, subjective well-being, anxiety, depression, transdiagnostic, youth

## Abstract

**Background/Objectives**: The relationship between experiential avoidance (EA) and anxiety and depression symptoms is well established, yet the adaptive psychological resources involved in these associations remain insufficiently clarified. This cross-sectional study tested a theoretically informed serial mediation model examining whether EA is associated with anxiety and depressive symptoms in a youth sample through lower spontaneity and subjective well-being. Creative adaptability was examined as an exploratory outcome of spontaneity. **Methods**: The sample comprised 444 participants aged 16–24 years (M = 18.87, SD = 2.04; 72.3% female), who completed self-report measures for EA, spontaneity, creative adaptability, subjective well-being, depressive and anxiety symptoms. Data were analyzed using IBM SPSS Statistics and Amos to estimate the observed-variable path model. **Results**: The model showed acceptable fit (CFI = 0.989, RMSEA = 0.074) and explained 54.1% of the variance in anxiety symptoms and 51.1% of the variance in depressive symptoms. EA was positively associated with depressive and anxiety symptoms (β = 0.531 and 0.448, *p* < 0.001) and negatively associated with spontaneity and subjective well-being (β = −0.441 and −0.250, *p* < 0.001). Spontaneity was positively associated with subjective well-being (β = 0.545) and creative adaptability (β = 0.384). The serial indirect effect from EA to symptoms through spontaneity and well-being was significant for both depression and anxiety (β = 0.089 and β = 0.088, *p* < 0.001). **Conclusions**: EA was associated with higher anxiety and depressive symptoms, and these associations were partly reflected in lower spontaneity and subjective well-being. In addition, spontaneity was associated with creative adaptability, although creative adaptability was not associated with symptom outcomes.

## 1. Introduction

Anxiety and depression are two of the most common mental health problems experienced by adolescents and young adults, representing a substantial and increasing global public health burden [1,2]. For instance, according to the global burden of disease analysis, the prevalence of anxiety and depression among youths ages 10–24 has risen gradually over time, with this increase being most notable between 2014 and 2021; the steepest rises were found for depressive symptoms in early adolescence and for anxiety disorders in the 20–24 age group [3].

These patterns have lasting developmental effects and have been linked to outcomes such as poor academic performance and social skills, poor quality of life, and increased likelihood of developing chronic mental health disorders in adulthood [4,5]. Thus, identifying psychological processes associated with internalizing symptoms in non-clinical youth should be considered a research priority, given their potential relevance for prevention.

Anxiety and depression frequently co-occur, both concurrently and sequentially [6]. Among youth with depression, anxiety comorbidity estimates range from 15% to 75% [7]. This comorbidity may be partly explained by symptom overlap, shared vulnerability factors such as negative affectivity and information-processing biases, and developmental pathways through which anxiety may increase the risk of later depressive symptoms [6,7]. Therefore, transdiagnostic approaches that target common etiological and maintenance mechanisms provide a relevant framework for understanding and treating internalizing problems in youth [8,9].

One transdiagnostic process associated with internalizing problems is experiential avoidance (EA), defined as the unwillingness to tolerate the presence of unpleasant mental states such as thoughts, feelings, memories, and bodily sensations combined with efforts to alter their form, frequency, or eliciting contexts, even when doing so undermines long-term adaptive functioning [10]. EA is placed within the broader framework of psychological inflexibility, emphasizing that avoidance becomes problematic when attempts to control internal experiences are excessive and rigid [11]. From a process perspective, EA can be seen as an emotion regulation strategy maintained through negative reinforcement, although avoidance may produce short-term relief [12]. This relief reinforces further avoidance and may gradually consolidate suppression, disengagement, and behavioral restriction. Over time, these patterns may narrow attention, reduce cognitive and behavioral flexibility, and maintain emotional arousal, perpetuating the internal states that avoidance was intended to reduce [11]. This process is particularly relevant to adolescence, when regulatory repertoires are still developing and early patterns of responding may have long-term effects.

The role of EA in psychopathology is well established. Meta-analytic evidence indicates moderate-to-large associations between EA and anxiety, depressive symptoms, obsessive-compulsive symptoms, and posttraumatic stress symptoms [13]. Longitudinal findings further indicate that EA may predict the persistence of major depressive disorder and generalized anxiety disorder in late adolescence, even after controlling for prior symptoms; by contrast, anxiety and depressive symptoms do not appear to predict later EA [14]. Evidence from adolescent samples further supports the clinical relevance of EA: in inpatient adolescents, EA has been associated with anxiety disorder independently of depressive symptoms [15], and prospective adolescent studies indicate that elevated EA predicts later anxiety and depressive symptoms after accounting for baseline symptom severity [16]. Thus, EA can be considered a theoretically and empirically relevant vulnerability process for internalizing symptoms in youth [17].

However, the intermediate processes linking EA with internalizing symptomatology remain insufficiently studied, particularly in youth samples. Although studies with adolescents and youth support the relevance of EA to anxiety and depressive symptomatology [15,16], existing research has focused predominantly on direct associations between EA and symptoms, with less attention to the adaptive psychological resources that may be reduced when individuals rely on avoidance. This gap is theoretically important because EA has been conceptualized as a transdiagnostic process relevant to depression and anxiety [10,13], implying that its associations may involve not only the maintenance of distress but also diminished positive emotional functioning and a narrower repertoire of flexible, context-sensitive responses [18,19,20].

Examining such intermediate processes may have practical relevance, as positive psychological resources could represent candidate targets for future prevention-oriented and intervention research. From this perspective, the association between EA and internalizing symptoms may partly reflect lower availability of adaptive resources, rather than a direct association with symptoms alone. Clarifying these intermediate associations is important because potentially modifiable positive resources may offer clinically useful targets for prevention and intervention [6,21].

Spontaneity is defined within Moreno’s psychodrama framework as a pre-creative, catalytic state of readiness that enables an adequate response to a new situation or a new response to an old one [22]. Adequacy refers to the contextual suitability, competence, and timeliness of the response, whereas novelty refers to its emergent, non-scripted and present-oriented character [23]. This conceptualization positions spontaneity in direct functional contrast to EA: whereas EA is characterized by rigidity and behavioral narrowing, spontaneity involves openness, flexibility, and adaptive engagement with present experience. On theoretical grounds, higher EA would be expected to be associated with lower spontaneous states, although to our knowledge, this association has not yet been directly tested. Spontaneity has been positively associated with attention to present behaviors, feelings, and thoughts [24] and negatively associated with emotional inhibition [25]. Evidence from adolescents and young adults further indicates that spontaneity is associated with higher well-being [26] and lower psychological distress, including lower anxiety and depressive symptoms [27]. Taken together, these findings support the relevance of spontaneity as a measurable and theoretically meaningful adaptive resource in adolescence and young adulthood.

Creative adaptability (CA) refers to the capacity to generate cognitive, emotional, and behavioral responses that are both personally new and potentially effective [28]. Conceptually, CA is closely aligned with Moreno’s view of creativity as essential for adaptive functioning [22] and may be understood as theoretically related to spontaneity. From this perspective, spontaneity reflects a state of readiness that can support flexible and creative responses to changing situational demands. Prior research has linked creative adaptability to resilience, adaptive coping, and emotional well-being, primarily in adult samples exposed to acute stressors or crisis contexts [29]. However, its role in the relation to EA and internalizing symptoms in youth has not been examined. Accordingly, in the present study, CA was treated as an exploratory auxiliary outcome of spontaneity, rather than as a central mediator in the proposed serial model.

Subjective well-being encompasses the cognitive evaluation of life satisfaction and the affective balance of positive over negative emotions [30]. Rather than being merely the absence of disorder, well-being constitutes a distinct positive dimension of mental health [31] and is consistently inversely associated with depressive and anxiety symptomatology [32,33]. For this reason, subjective well-being was positioned as a proximal positive mental-health outcome linking reduced adaptive functioning with higher internalizing symptoms.

Integrating these constructs, we aim to test a theoretically informed serial mediation model in which EA is hypothesized to be associated with internalizing symptoms through lower spontaneity and lower subjective well-being. This sequence was grounded in evidence identifying EA as a transdiagnostic process linked to anxiety and depressive symptoms [13,34], and in the assumption that avoidant regulation may restrict engagement with valued activities and present-moment experience [35], thereby limiting spontaneous, flexible responding and undermining subjective well-being. Spontaneity, in turn, has been conceptualized as a readiness for adaptive and present-oriented responding [22,23], and empirical findings indicate that it is positively associated with attention to present behaviors, feelings, and thoughts [24], negatively associated with emotional inhibition [25], anxiety and depression symptoms [27], and positively related to well-being and lower stress [36]. Finally, lower well-being has been linked to higher internalizing symptoms [32].

The proposed serial ordering, spontaneity preceding subjective well-being, is grounded in Moreno’s framework, which conceptualizes spontaneity as a pro-creative state of readiness enabling adequate and novel responses to situational demands, rather than an evaluative appraisal of circumstances. Subjective well-being, by contrast, reflects a cognitive evaluation of life satisfaction and affective balance [30], presupposing prior engagement with experience. Spontaneity is therefore theoretically and statistically specified as preceding well-being in the hypothesized model, a position conceptually consistent with findings linking higher spontaneity to well-being in youth samples [32,36]. Thus, the proposed sequence represents a theory-driven explanatory model that was statistically tested in the present study.

Six hypotheses were derived from this framework:

**H1.**  *Experiential avoidance is negatively associated with spontaneity and subjective well-being.*

**H2.**  *Experiential avoidance is positively associated with depressive and anxiety symptoms.*

**H3.**  *Spontaneity is positively associated with subjective well-being.*

**H4.**  *Spontaneity is positively associated with creative adaptability, on an exploratory basis given its theoretical proximity.*

**H5.**  *Subjective well-being is negatively associated with depressive and anxiety symptoms.*

**H6.**  *Spontaneity and subjective well-being will serially mediate the relationship between experiential avoidance and internalizing symptoms.*

## 2. Materials and Methods

### 2.1. Study Design and Participants

The present study used a cross-sectional correlational design. Participants were youths aged 16–24 years, recruited from high schools and several university programs. Data were collected between October and November 2025 through an online questionnaire administered via Google Forms. For participants under 18 years of age, legal guardian consent was obtained before participation. All participants were informed about data processing and use, confidentiality, and their right to withdraw from the study at any time. All questionnaire items were mandatory; therefore, no item-level missing data were present. No incentives were offered.

Inclusion criteria were age between 16 and 24 years and provision of informed consent. No diagnostic screening procedures or mental-health-related exclusion criteria were applied. Thus, the sample was non-clinical by recruitment source rather than by symptom presentation. Because the survey link was distributed through educational settings without a fixed sampling frame, a formal response rate could not be calculated. A total of 450 participants completed the questionnaire. Six respondents failed the attention-check item and were excluded, resulting in a final analytic sample of 444 participants.

An a priori power analysis conducted using G*Power 3.1 [37] indicated that a minimum of 119 participants was required to detect a medium effect size (*f*^2^ = 0.15) with α = 0.05, power = 0.95 and three predictors.

The final sample exceeded this requirement and, in the context of the observed-variable path model, corresponds to approximately 21 observations per freely estimated parameter, above the recommended 10:1 ratio [38].

The final sample comprised 444 participants, with an age range from 16 to 24 years (M = 18.87, SD = 2.04) of whom 72.3% (*n* = 321) identified as female, 26.8% (*n* = 119) as male, and 0.9% (*n* = 4) as another gender category. In terms of educational level, 49.8% (*n* = 221) were high school students, 0.2% (*n* = 1) attended vocational school, 43.7% (*n* = 194) were undergraduate students, 6.1% (*n* = 27) were graduate students, and 0.2% (*n* = 1) were not currently enrolled in any form of education. Most participants reported living in urban areas, 70.3% (*n* = 312), compared with 29.7% (*n* = 132) in rural areas.

### 2.2. Measures

Demographic Questionnaire. Participants completed a short demographic questionnaire regarding age, gender, educational status, and area of residence.

Experiential Avoidance. Experiential avoidance was measured using the Acceptance and Action Questionnaire-II (AAQ-II) [10]. The AAQ-II is a 7-item self-report that measures experiential avoidance. Items are rated on a 7-point Likert scale ranging from 1 (never true) to 7 (always true), with higher scores indicating greater levels of experiential avoidance. The Romanian version showed preliminary evidence of adequate internal consistency, with a Cronbach’s α of 0.84 [39]. In the present study, internal consistency, as indicated by Cronbach’s alpha, was α = 0.90.

Spontaneity. Spontaneity was measured using the Revised Spontaneity Assessment Inventory (SAI-R) [40]. The SAI-R assesses the occurrence of spontaneous states; participants indicated the intensity with which they typically experience different spontaneous states in everyday life. The inventory consists of 18 items rated on a 5-point Likert scale ranging from 1 (very weak) to 5 (very strong). Total scores were calculated by summing item responses, with higher scores indicating higher spontaneity. The Cronbach’s alpha coefficient for the SAI-R in previous youth studies was 0.86 [26]. As no validated Romanian version of the SAI-R was available, the scale was translated and culturally adapted using a forward–backward method. In the present study, internal consistency coefficient, as indicated by Cronbach’s alpha, was α = 0.90.

Creative Adaptability. Creative adaptability was measured using the Creative Adaptability Scale (CAS) [28]. The scale measures participants’ cognitive, behavioral, and emotional ability to respond creatively and adaptively in stressful or changing situations. The scale includes 9 items, rated on a 5-point scale from 1 (not at all like me) to 5 (very much like me). Total scores were calculated by summing item responses. The scale has demonstrated good psychometric properties with Cronbach’s alpha for the CAS total score of 0.90 [28]. Because no validated Romanian version of the CAS was available, the scale was translated and culturally adapted for the purposes of the present study using a forward–backward translation method. In the present sample, the Cronbach’s alpha coefficient for internal consistency was α = 0.88.

Well-Being. Well-being was assessed using the World Health Organization-Five Well-Being Index (WHO-5). The WHO-5 is a brief self-report measure of subjective well-being over the previous two weeks, including positive mood, vitality, interest, and general functioning. The scale includes 5 items rated on a 6-point Likert scale ranging from 0 (at no time) to 5 (all the time), with higher scores indicating higher subjective well-being. Following the Romanian scoring instructions, item scores were summed and multiplied by four, resulting in a total score ranging from 0 to 100. The WHO-5 has demonstrated sound psychometric properties, including good reliability and measurement invariance across multiple countries and populations [41]. In the present study, internal consistency was α = 0.89.

Depressive Symptoms. Depressive symptoms were assessed using the Patient Health Questionnaire-9 (PHQ-9) [42]. The PHQ-9 is a 9-item self-report instrument that assesses depressive symptoms experienced during the previous two weeks. Items are rated on a 4-point Likert scale ranging from 0 (not at all) to 3 (nearly every day). Total scores were calculated by summing item responses, with higher scores indicating higher depressive symptom severity. The PHQ-9 has been cross-culturally adapted and validated in a Romanian sample, showing good internal consistency, test–retest reliability, and convergent validity [43]. The internal consistency coefficient, as indicated by Cronbach’s alpha, was 0.87.

Anxiety Symptoms. Anxiety symptoms were assessed using the Generalized Anxiety Disorder-7 scale (GAD-7) [44]. The GAD-7 is a 7-item self-report measure assessing anxiety symptoms experienced during the previous two weeks. Items are rated on a 4-point Likert scale ranging from 0 (not at all) to 3 (nearly every day). Total scores were calculated by summing item responses, with possible scores ranging from 0 to 21, with higher scores indicating higher anxiety symptom severity. The Romanian adaptation of the GAD-7 has shown satisfactory internal consistency, an acceptable factor structure, and convergent validity in both clinical and non-clinical Romanian samples [45]. The Cronbach’s alpha coefficient for internal consistency was α = 0.88.

### 2.3. Statistical Analysis

Data were analyzed using IBM SPSS Statistics version 26, IBM SPSS Amos version 18, and the PROCESS macro for SPSS (version 5.0). Data were first screened for missing values, outliers, data entry errors, and consistency between item responses and the theoretical score ranges of the instruments. Normality was evaluated using skewness and kurtosis values, together with visual inspection of histograms and Q–Q plots. Given the sensitivity of formal normality tests in larger samples, these indicators were prioritized. Following Kim’s recommendations for samples larger than 300, no substantial departure from normality was assumed when absolute skewness and kurtosis values were within acceptable limits [46].

The hypothesized model was tested as an observed-variable path using maximum likelihood estimation. EA was specified as the exogenous predictor in the statistical model; spontaneity and subjective well-being were specified as serial mediators; and depressive and anxiety symptoms were specified as endogenous outcomes. CA was included as an exploratory endogenous outcome associated with spontaneity. The residual covariance between PHQ-9 and GAD-7 was freely estimated, given the strong association between depressive and anxiety symptoms.

Model fit was evaluated using Model Chi-Square (χ^2^) and the Chi-Square to degrees of freedom ratio (χ^2^/df), Root Mean Square Error of Approximation (RMSEA), Comparative Fit Index (CFI), Tucker–Lewis Index (TLI), and Probability of Close Fit (PCLOSE). Because χ^2^ is sensitive to sample size, model evaluation relied primarily on approximate fit indices. CFI and TLI values ≥ 0.95, RMSEA values ≤ 0.08, and PCLOSE values > 0.05 were considered indicative of acceptable to good fit [47]. Given that the upper bound of the RMSEA 90% confidence interval can exceed 0.08 in well-fitting models with few degrees of freedom [47], model evaluation relied on the convergent pattern of multiple fit indices rather than any single criterion.

Indirect effects in the Amos model were tested using 5000 bias-corrected bootstrap samples and 95% confidence intervals. Because the standard Amos output used in the present analysis provided total indirect effects but did not directly decompose path-specific indirect effects for the serial mediation chain, supplementary regression-based mediation analyses were conducted using Hayes’s PROCESS macro for SPSS, Model 6 [48], with 5000 bootstrap samples and 95% bias-corrected confidence intervals. These analyses were used only to decompose specific indirect pathways and were interpreted alongside the primary Amos path model.

As a sensitivity analysis, the primary model was re-estimated with age and gender included as covariates predicting spontaneity, subjective well-being, depressive symptoms, and anxiety symptoms. Because demographic moderation was not a primary aim and the study was not specifically powered for interaction testing, this analysis was interpreted as a robustness check rather than as a formal moderation analysis.

Common method variance was assessed using Harman’s single-factor test and a single-factor CFA in Amos, with all items specified as indicators of one general latent factor [49].

### 2.4. Ethical Approval

The present study was conducted in accordance with the Declaration of Helsinki, and it was approved by the Ethical Committee of Alexandru Ioan Cuza University of Iași, Romania (approval no. 1131/18 June 2025). Before completing the online survey, participants were informed about the purpose of the study, the use of the data, the confidentiality of their responses, and the voluntary nature of participation. Informed consent was obtained from all participants prior to data collection, and for participants under 18 years of age, written consent was also obtained from a parent or legal guardian. No financial or material incentives were provided.

## 3. Results

### 3.1. Descriptive Statistics and Correlational Analyses

Table 1 presents the descriptive statistics, distributional indices, and Pearson correlations for all study variables. The distributions of the variables were examined using skewness and kurtosis values, together with visual inspection of histograms and Q–Q plots. Skewness values ranged from −0.25 to 0.53, and excess kurtosis values ranged from −0.80 to −0.05, indicating no substantial departure from univariate normality [46].

Pearson’s correlation analysis revealed a positive and statistically significant association of experiential avoidance with anxiety (*r* = 0.68, *p* < 0.01) and depressive symptoms (*r* = 0.63, *p* < 0.01), and a negative association with spontaneity (*r* = −0.44, *p* < 0.01) and well-being (*r* = −0.49, *p* < 0.01). The association between experiential avoidance and creative adaptability was not statistically significant (r = −0.05, *p* > 0.05). Spontaneity was positively and significantly associated with creative adaptability (*r* = 0.38, *p* < 0.01) and well-being (*r* = 0.66, *p* < 0.01) and negatively associated with anxiety (*r* = −0.39, *p* < 0.01) and depressive symptoms (*r* = −0.46, *p* < 0.01). Creative adaptability was positively and significantly associated with well-being (*r* = 0.18, *p* < 0.01) and not significantly associated with anxiety (*r* = −0.05, *p* > 0.05) or depressive symptoms (*r* = −0.06, *p* > 0.05). Well-being was negatively and significantly associated with anxiety (*r* = −0.57, *p* < 0.01) and depressive symptoms (*r* = −0.60, *p* < 0.01). Anxiety and depressive symptoms were strongly correlated (*r* = 0.77, *p* < 0.01).

Furthermore, to provide additional descriptive statistics regarding the sample, we examined the proportion of participants with symptom scores at or above conventional screening thresholds. Based on the standard cut-off of ≥10, 220 participants (49.5%) met or exceeded the PHQ-9 screening threshold for depressive symptoms, whereas 195 participants (43.9%) met or exceeded the GAD-7 screening threshold for anxiety symptoms.

### 3.2. Common Method Variance Assessment

To assess potential common method variance, we conducted Harman’s single-factor test and a single-factor CFA. Harman’s test indicated that the first unrotated component explained 29.03% of the total variance, below the commonly used 50% criterion for a dominant general factor [49]. In addition, the single-factor CFA showed poor fit, χ^2^(1430) = 8036.23, *p* < 0.001, χ^2^/*df* = 5.62, CFI = 0.516, TLI = 0.497, RMSEA = 0.102, 90% CI [0.100, 0.104]. These findings suggest that the observed associations were unlikely to be primarily attributable to a single common method factor.

### 3.3. Serial Mediation Model

The serial mediation model was tested using SEM with maximum likelihood estimation in IBM SPSS AMOS, Version 18. EA was specified as the exogenous predictor in the statistical model, spontaneity and subjective well-being were specified as serial mediators, and anxiety and depressive symptoms were specified as endogenous outcomes. Spontaneity was also specified as a predictor of CA (H4). The residual covariance between the anxiety and depressive symptom error terms was freely estimated to account for their high correlation. Supplementary PROCESS analyses were used to decompose path-specific indirect effects and were interpreted alongside the primary Amos path model.

#### 3.3.1. Model Fit

**Figure 1:**
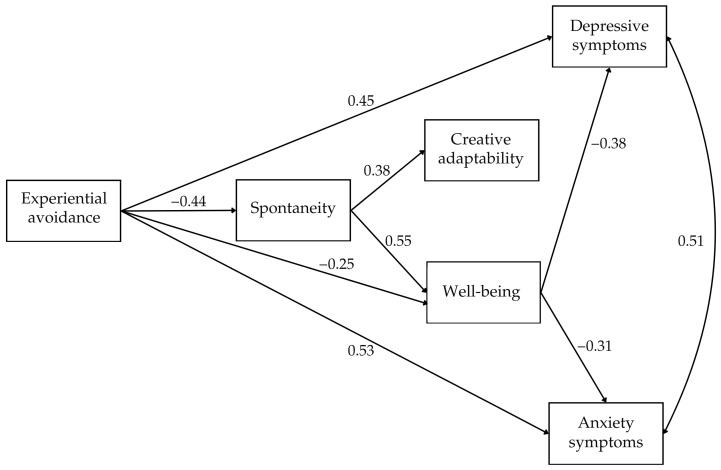
The hypothesized serial mediation model with standardized path coefficients. Single-headed arrows indicate specified regression paths; the curved double-headed arrow indicates the residual covariance between depressive and anxiety symptoms. All displayed paths were statistically significant at *p* < 0.001.

The SEM model demonstrated acceptable fit to the data. The Chi-square test was χ^2^(6) = 20.46, *p* = 0.002, χ^2^/*df* = 3.41; the significant χ^2^ statistic was expected given the sample size and should not be interpreted in isolation [47]. The incremental fit indices were CFI = 0.989 and TLI = 0.971, both exceeding the 0.95 threshold. The RMSEA point estimate was 0.074 (90% CI [0.040, 0.110]), with PCLOSE = 0.112. Although the upper bound of the RMSEA confidence interval (0.110) exceeds the 0.10 threshold for poor fit, this imprecision reflects the low degrees of freedom (*df* = 6) of the observed-variable path model rather than substantive misfit [47]; the point estimate is below 0.08 and the remaining fit indices consistently support model adequacy. The model accounted for 19.5% of the variance in spontaneity, 48.0% in well-being, 54.1% in anxiety symptoms, 51.1% in depressive symptoms, and 14.8% in creative adaptability. The final model with standardized path coefficients is presented in Figure 1.

An alternative theoretically plausible model positioning well-being before spontaneity was also tested. However, this alternative structure showed poor model fit, χ^2^(7) = 107.24, *p* < 0.001, χ^2^/*df* = 15.32, CFI = 0.920, TLI = 0.830, RMSEA = 0.180, 90% CI [0.151, 0.211], AIC = 147.24, and demonstrated poorer fit compared to the hypothesized model. These findings support the plausibility of retaining the proposed statistical specification in the present cross-sectional data but should not be interpreted as confirming the temporal ordering of the variables.

#### 3.3.2. Direct Effects

The unstandardized and standardized coefficients for all the paths are presented in Table 2. Experiential avoidance was negatively associated with spontaneity (β = −0.441, *p* < 0.001) and well-being (β = −0.250, *p* < 0.001), supporting H1, and positively associated with anxiety (β = 0.531, *p* < 0.001) and depressive symptoms (β = 0.448, *p* < 0.001), supporting H2. Spontaneity was positively associated with well-being (β = 0.545, *p* < 0.001), supporting H3, and creative adaptability (β = 0.384, *p* < 0.001), supporting H4. Well-being was negatively associated with anxiety symptoms (β = −0.312, *p* < 0.001) and depressive symptoms (β = −0.379, *p* < 0.001), supporting H5.

#### 3.3.3. Indirect Effects

Regarding the total indirect effects presented in Table 3, experiential avoidance had a significant negative total indirect effect on subjective well-being, *B* = −0.519, 95% BC CI [−0.653, −0.387], β = −0.241, 95% BC CI [−0.300, −0.184] and also significant positive total indirect effects on anxiety symptoms, *B* = 0.083, 95% BC CI [0.058, 0.112], β = 0.153, 95% BC CI [0.108, 0.202], and depressive symptoms, *B* = 0.116, 95% BC CI [0.085, 0.152], β = 0.186, 95% BC CI [0.138, 0.239]. The indirect effect of experiential avoidance on creative adaptability through spontaneity was also significant, *B* = −0.116, 95% BC CI [−0.155, −0.083], β = −0.170, 95% BC CI [−0.221, −0.122].

Supplementary PROCESS analyses using Model 6 with 5000 bootstrap samples were conducted to decompose path-specific indirect effects in the serial mediation chain. These analyses estimated the specific indirect effects from experiential avoidance to symptom outcomes through spontaneity alone, subjective well-being alone, and the serial pathway involving spontaneity and subjective well-being.

As reported in Table 4, the serial-specific indirect effect from experiential avoidance to depressive symptoms through spontaneity and subjective well-being was significant, *B* = 0.056, 95% bootstrap CI [0.036, 0.081], β = 0.089. The corresponding serial-specific indirect effect for anxiety symptoms was also significant, *B* = 0.048, 95% bootstrap CI [0.031, 0.068], β = 0.088.

These findings were consistent with H6, as the serial indirect effects through spontaneity and subjective well-being were significant for both depressive and anxiety symptoms. Importantly, the PROCESS estimates represent path-specific components of the total indirect effects reported in Table 3. Therefore, the AMOS and PROCESS results should be interpreted as complementary rather than as independent analytic models.

#### 3.3.4. Sensitivity Analysis

A sensitivity analysis was conducted by re-estimating the primary path model with age and gender included as covariates. For this analysis, gender was dummy-coded as 0 = female and 1 = male or another gender category. The covariate-adjusted model showed acceptable fit, χ^2^ (8, *n* = 444) = 20.58, *p* = 0.008, CFI = 0.990, TLI = 0.966, RMSEA = 0.060, 90% CI [0.028, 0.092], PCLOSE = 0.271. The direction, magnitude, and statistical significance of the primary paths remained stable: EA → spontaneity, β = −0.431; EA → well-being, β = −0.232; EA → anxiety symptoms, β = 0.529; EA → depression symptoms, β = 0.454; spontaneity → well-being, β = 0.531; spontaneity → CA, β = 0.384; well-being → anxiety symptoms, β = −0.299; and well-being → depression symptoms, β = −0.371, *p* < 0.001. The indirect effects from EA to well-being, anxiety, depression, and CA also remained statistically significant, with bias-corrected 95% confidence intervals not including zero. Among the covariates, gender was significantly associated with well-being, β = 0.145, *p* < 0.001, indicating higher well-being scores among participants coded as male or another gender category relative to those coded as female. Age was weakly negatively associated with depressive symptoms, β = −0.080, *p* = 0.011. The remaining direct covariate paths were nonsignificant. Overall, the main pattern of associations was not materially altered after adjusting for age and dummy-coded gender.

## 4. Discussion

This study examined the association between EA and depressive and anxiety symptoms in a youth sample through a theoretically informed serial model involving spontaneity and subjective well-being. The findings showed that higher EA was associated with higher depressive and anxiety symptoms and with lower spontaneity and well-being. Spontaneity was positively associated with well-being and CA, whereas well-being was negatively associated with both symptom outcomes. The serial indirect associations from EA to depressive and anxiety symptoms through spontaneity and well-being were statistically significant. At the same time, the direct associations between EA and depressive and anxiety symptoms remained the strongest effects in the model, whereas the indirect associations through spontaneity and subjective well-being were statistically reliable but modest. Thus, spontaneity and well-being should be viewed as statistically associated mediators and candidate adaptative resources for future longitudinal and intervention research, rather than as demonstrated causal mechanisms.

The associations between EA and both depressive and anxiety symptoms are consistent with the broader transdiagnostic literature. Meta-analytic evidence indicates moderate-to-large associations between EA and symptoms of depression and anxiety [13], and longitudinal findings suggest that EA predicts depressive and anxiety symptoms in adolescent and young adult samples [14,16]. The present findings are consistent with this literature and extend it by examining whether positive adaptive resources may be involved in these associations.

Regarding spontaneity, the findings showed a significant negative association between EA and spontaneity. This result is theoretically coherent with Moreno’s framework, in which spontaneity is understood as a state of readiness that enables new and adequate responses, whereas EA is described as its functional opposite [22,23]. Because EA involves rigid attempts to control or avoid internal experience and may narrow the behavioral repertoire [10,12], higher EA would be expected to co-occur with lower spontaneity. The present findings are consistent with this account and, to our knowledge, provide the first direct test of the association between EA and spontaneity in adolescents and young adults. Although previous studies have examined spontaneity in relation to psychological distress [36], its association with EA had not yet been directly tested.

Spontaneity was also positively associated with well-being, and well-being was negatively associated with depressive and anxiety symptoms. These findings align with previous evidence showing that spontaneity is associated with higher well-being in adolescents and young adults [26,36], and that well-being is inversely related to internalizing symptoms [31,33]. In the tested model, the serial indirect association from EA to symptom outcomes through spontaneity and well-being was statistically significant, suggesting that part of the association between EA and symptoms was reflected in lower spontaneity and lower well-being. This interpretation remains preliminary given the cross-sectional design.

An important issue concerns the empirical separability of the model components. The correlation pattern suggested two strongly interrelated clusters: EA, anxiety, and depression on one side, and spontaneity and well-being on the other. This pattern may reflect substantive links between maladaptive and adaptive psychological functioning, but it may also be influenced by measurement overlap. For example, the AAQ-II has been criticized for overlap with negative affect [50], and the WHO-5 may share content with depressive symptom measures [51]. Therefore, the direct and indirect associations observed in the model may partly reflect shared affective content rather than fully distinct psychological processes.

Although spontaneity and well-being were both implicated in the serial statistical model, their conceptual distinctiveness remains important. Within Moreno’s framework, spontaneity refers to a present-oriented state of readiness for new and adequate responses [22,23], whereas well-being reflects a broader evaluative appraisal of life satisfaction, vitality, and affective balance [31,43]. In the present results, the strong association between these constructs is consistent with their theoretical proximity, but it also raises questions about their empirical separability in cross-sectional self-report designs. Future studies using latent-variable models, longitudinal designs, and multi-method assessment are needed to determine whether spontaneity and well-being operate as distinct processes over time.

CA was examined as an exploratory outcome given its theoretical proximity to spontaneity. The results showed that spontaneity was positively associated with CA, and EA was indirectly associated with CA through spontaneity. These findings are consistent with the theory stating that spontaneity may support creative-adaptive responses [22,28]. However, CA was not directly associated with EA, depressive symptoms, or anxiety symptoms in this sample. Thus, within the current model, CA should be interpreted primarily as an auxiliary outcome supporting the spontaneity–creativity framework, rather than as a central explanatory component of internalizing symptoms, particularly under conditions of stress, contextual change, or intervention.

Additional analyses supported the robustness and theoretical plausibility of the proposed model. The sensitivity analysis showed that the main associations remained stable after adjusting for age and gender. In addition, an alternative statistical model positioning well-being before spontaneity showed poor fit, supporting the plausibility of the proposed model specification. However, this comparison should not be interpreted as evidence that the proposed temporal sequence is confirmed, and the findings should be interpreted as statistical mediation rather than as evidence of temporal or causal processes.

### 4.1. Limitations

Several limitations qualify these conclusions. First, the cross-sectional design precludes causal or temporal inferences. Therefore, the serial indirect effects should be interpreted strictly as statistical mediation: evidence consistent with the proposed theoretical model and useful as a basis for future longitudinal testing, but not as evidence of causal effects, temporal ordering, or directional processes among the variables.

Second, measurement overlap and discriminant validity represent a major interpretive limitation. The AAQ-II may partly capture general distress or negative affectivity, while the WHO-5 may overlap inversely with depressive symptom content. Thus, both the direct and indirect associations may have been strengthened by shared affective content. Future studies should use latent-variable approaches, alternative measures, and multi-method assessments to test whether these constructs remain empirically distinct.

Third, all variables were assessed through self-report at a single time point. Although Harman’s single-factor test and the single-factor CFA did not support a dominant common method factor, common method variance cannot be fully ruled out. Future studies should address this through procedural approaches (e.g., temporal separation of measurements, multi-informant designs, experience-sampling methodology) and statistical approaches (e.g., latent method factor in CFA).

Fourth, the Romanian versions of the SAI-R and CASs were translated and back-translated for this study but have not yet undergone full psychometric validation. Although internal consistency was good, reliability alone does not establish construct validity or cross-cultural equivalence. Future studies should examine factor structure, convergent and discriminant validity, test–retest reliability, and measurement invariance, and should consider state-based measures of spontaneity.

Fifth, the convenience sample was predominantly female, recruited from a single cultural context, and included both adolescents and emerging adults. These developmental differences may influence the associations examined in this study. Although age was included as a covariate in the sensitivity analysis, the study was not powered to formally test developmental subgroup moderation. Moreover, the sample was non-clinical by recruitment source rather than symptom presentation. As reported in Results section, nearly half of participants scored at or above clinical screening thresholds for depressive and anxiety symptoms; generalization to clinically diagnosed populations and low-symptom community samples should therefore be made with caution.

Finally, the study did not assess alternative mediators, such as rumination, worry, expressive suppression, or broader emotion regulation difficulties. In addition, the specific indirect effects were estimated separately from the primary SEM model and should therefore be interpreted as complementary rather than fully equivalent to the path model estimates.

### 4.2. Implications and Future Research

The study provides preliminary evidence that spontaneity can be situated within a theoretically informed model linking EA, well-being, and internalizing symptoms. Future research should first test the temporal ordering of the proposed model, specifically whether EA precedes lower spontaneity, whether lower spontaneity precedes reduced subjective well-being, and whether these processes are subsequently associated with higher anxiety and depressive symptoms.

Longitudinal, experimental, and experience-sampling designs are needed to clarify directionality, distinguish spontaneity and well-being as potentially separable candidate processes, and reduce measurement overlap among EA, well-being, and symptom constructs. State-based, behavioral, observer-rated, and multi-method assessments would further strengthen the empirical evaluation of spontaneity as a potentially modifiable protective resource.

## 5. Conclusions

The present study provides preliminary evidence that experiential avoidance is associated with higher depressive and anxiety symptoms in youth, and that these associations are statistically linked to lower spontaneity and lower subjective well-being. The findings support the theoretical plausibility of a model in which reduced adaptive psychological resources accompany higher experiential avoidance and internalizing symptoms.

Spontaneity and subjective well-being emerged as relevant positive psychological resources within the model. Spontaneity was negatively associated with experiential avoidance and positively associated with well-being, in line with the view that it reflects a readiness for flexible and contextually appropriate responding. Subjective well-being, in turn, was negatively associated with depressive and anxiety symptoms, suggesting its relevance as a positive mental-health correlate in this sample. By contrast, creative adaptability was associated with spontaneity but showed no direct associations with experiential avoidance, depressive symptoms, or anxiety symptoms. Accordingly, in the current study, creative adaptability should be interpreted as an exploratory outcome supporting the spontaneity–creativity framework, rather than as a central explanatory component of internalizing symptoms.

Overall, the findings extend transdiagnostic perspectives on experiential avoidance, subjective well-being, and youth internalizing symptoms by incorporating spontaneity and creative adaptability, two psychodrama-informed constructs. In this context, spontaneity, subjective well-being, and creative adaptability should all be considered relevant candidates for future longitudinal and experimental studies.

## Figures and Tables

**Table 1:** Descriptive statistics and Pearson correlations among study variables.

Variable	M	SD	Skewness	Kurtosis	1	2	3	4	5
1. Experiential Avoidance	23.92	10.02	0.52	−0.57	—				
2. Spontaneity	64.20	11.08	−0.25	−0.06	−0.44 **	—			
3. Creative Adaptability	29.08	6.86	−0.15	−0.05	−0.05	0.38 **	—		
4. Subjective Well-Being	55.89	21.59	−0.09	−0.64	−0.49 **	0.66 **	0.18 **	—	
5. Anxiety Symptoms	9.39	5.47	0.36	−0.80	0.68 **	−0.39 **	−0.05	−0.57 **	—
6. Depressive Symptoms	10.16	6.26	0.53	−0.39	0.63 **	−0.46 **	−0.06	−0.60 **	0.77 **

Note. *N* = 444 for all variables and all pairwise correlations; no item-level missing data were present. ** *p* < 0.01.

**Table 2:** Direct effects in the serial mediation model.

Path	B	SE (B)	95% BC CI for B	β	95% BC CI for β	*p*
EA → Spontaneity	−0.488	0.047	[−0.595, −0.372]	−0.441	[−0.525, −0.344]	<0.001
EA → Subjective Well-Being	−0.539	0.082	[−0.724, −0.361]	−0.250	[−0.333, −0.169]	<0.001
EA → Anxiety Symptoms	0.290	0.020	[0.246, 0.334]	0.531	[0.450, 0.605]	<0.001
EA → Depressive Symptoms	0.280	0.024	[0.230, 0.333]	0.448	[0.372, 0.522]	<0.001
Spontaneity → Subjective Well-Being	1.062	0.074	[0.909, 1.204]	0.545	[0.464, 0.612]	<0.001
Spontaneity → Creative Adaptability	0.238	0.027	[0.179, 0.293]	0.384	[0.289, 0.467]	<0.001
Well-Being → Anxiety Symptoms	−0.079	0.009	[−0.099, −0.058]	−0.312	[−0.389, −0.229]	<0.001
Well-Being → Depressive Symptoms	−0.110	0.011	[−0.132, −0.085]	−0.379	[−0.453, −0.294]	<0.001

Note. EA = experiential avoidance; B = unstandardized regression coefficient; SE (B) = standard error of the unstandardized coefficient; β = standardized regression coefficient; BC CI = bias-corrected bootstrap confidence interval based on 5000 bootstrap samples.

**Table 3:** Total indirect effects (Amos Path Model).

Indirect Effect	B	SE (B)	95% BC CI for B	β	95% BC CI for β
EA → Subjective Well-Being	−0.519	0.069	[−0.653, −0.387]	−0.241	[−0.300, −0.184]
EA → Anxiety Symptoms	0.083	0.014	[0.058, 0.112]	0.153	[0.108, 0.202]
EA → Depressive Symptoms	0.116	0.017	[0.085, 0.152]	0.186	[0.138, 0.239]
EA → Creative Adaptability	−0.116	0.018	[−0.155, −0.083]	−0.170	[−0.221, −0.122]

Note. EA = experiential avoidance; B = unstandardized indirect effect; SE (B) = bootstrap standard error; β = standardized indirect effect; BC CI = bias-corrected bootstrap confidence interval based on 5000 bootstrap samples. Confidence intervals that do not include zero indicate statistical significance.

**Table 4:** Specific indirect effects estimated using PROCESS Model 6.

Specific Indirect Effect	B	Boot SE	95% Bootstrap CI for B	β
EA → Spontaneity → Depressive Symptoms	0.005	0.013	[−0.021, 0.030]	0.007
EA → Subjective Well-Being → Depressive Symptoms	0.058	0.013	[0.035, 0.085]	0.092
EA → Spontaneity → Subjective Well-Being → Depressive Symptoms	0.056	0.011	[0.036, 0.081]	0.089
EA → Spontaneity → Anxiety Symptoms	−0.023	0.013	[−0.049, −0.001]	−0.042
EA → Subjective Well-Being → Anxiety Symptoms	0.050	0.011	[0.031, 0.072]	0.092
EA → Spontaneity → Subjective Well-Being → Anxiety Symptoms	0.048	0.010	[0.031, 0.068]	0.088

Note. EA = experiential avoidance; B = unstandardized specific indirect effect; Boot SE = bootstrap standard error; β = completely standardized specific indirect effect; CI = confidence interval based on 5000 bootstrap samples. Confidence intervals that do not include zero indicate statistical significance.

## Data Availability

Data are available upon request from the corresponding author.
